# Cost of Oncology Drugs in the Middle-Eastern Country of Lebanon: An Update (2014-2016)

**DOI:** 10.1200/JGO.17.00179

**Published:** 2018-04-02

**Authors:** Fadia Elias, Ibrahim R. Bou-Orm, Salim M. Adib, Selim Gebran, Anthony Gebran, Walid Ammar

**Affiliations:** **Fadia Elias**, **Ibrahim R. Bou-Orm**, and **Walid Ammar**, Ministry of Public Health, Lebanon; **Fadia Elias** and **Walid Ammar**, Lebanese University; **Salim M. Adib**, **Selim Gebran**, **Anthony Gebran**, and **Walid Ammar**, American University of Beirut, Beirut, Lebanon; and **Ibrahim R. Bou-Orm**, Queen Margaret University, Edinburgh, United Kingdom.

## Abstract

**Purpose:**

This study aims to evaluate trends in the increasing costs of oncology drugs procured by the Lebanese Ministry of Public Health (MOPH) between 2014 and 2016 and to assess the impact of the introduction in mid-2015 of new immunotherapy drugs for the treatment of lung cancer on the overall and specific costs of that treatment.

**Methods:**

A secondary analysis of data from the MOPH Cancer Drug Scientific Committee data base was conducted using a total of 18,133 cancer files between 2014 and 2016.

**Results:**

Over the 3-year period, about $140 million (USD) was spent on cancer drugs by the MOPH free cancer drug dispensing program. The expenditures increased by 27% after immunotherapy was phased in. The average cost of drugs per patient per year measured across all cancer types increased from $7,000 in 2014 to $8,400 in 2016. Trastuzumab, approved for treating human epidermal growth factor receptor 2–positive breast cancer ranked first in total expenditures for 2014-2015. By 2016, two new immunotherapy drugs had topped the list: pembrolizumab ranked first and nivolumab ranked third, representing 64% of the total cost of lung cancer treatment and approximately 19% of the total yearly budget; beneficiaries represented only 3% of all patients.

**Conclusion:**

This update documents the increasing financial impact of newer cancer drugs on the procurement process in the middle-income country of Lebanon. The trend is aligned with the financial burden of cancer drugs worldwide, which calls for a collaborative global response to this crisis.

## INTRODUCTION

Lebanon is facing a mounting financial and health care burden from cancer, which doubled in incidence in the last 50 years and is associated primarily with the steady aging of the population.^1,2^ To avoid catastrophic financial consequences for households as a result of out-of-pocket expenditures for cancer treatment, the Ministry of Public Health (MOPH) has been providing free oncology drugs since 1999 to all Lebanese patients who have no formal health coverage; almost half the Lebanese population of about 4.3 million are in this category.^3^ A previous assessment of MOPH data between 2008 and 2013 showed a steady increase in the average costs of cancer drugs per patient from $4,863 to $7,803 (note that US dollars are used throughout). Expensive targeted therapy has been approved by the MOPH for treating the majority of cancer types for several years.^4^ More recently, in 2015, immunotherapy was also approved for treating some types of cancer. Although this advance has a major positive impact on patients’ survival, it brings an additional devastating economic burden to the population and the health care system.^5^

This article updates the previously published figures on the costs of cancer drugs incurred by the Lebanese MOPH from 2014 to 2016. It specifically assesses the impact of the introduction in mid-2015 of two new immunotherapy drugs for treating lung cancer, pembrolizumab (Keytruda) and nivolumab (Opdivo), on the overall cost of cancer drugs and the specific cost of drugs for lung cancer.

## METHODS

This is a secondary analysis of data from the MOPH Cancer Drug Scientific Committee data base. A total of 18,133 cancer files on patients who had received approval for drug treatment coverage for years 2014 to 2016 were included in the analysis. The researchers analyzed de-identified data. The total cost of oncology drugs as well as the average cost per patient (measured by dividing the total annual cost by the total number of patients with cancer) were computed for each year. Expenditures by drug type were tabulated for the most expensive drugs. The changes in prices for selected cancer drugs for two time periods (2011 to 2013 and 2013 to 2015) were graphed to assess the impact of MOPH policies on the pricing of these drugs.

## RESULTS

### Total Financial Burden of Cancer Drugs Incurred by MOPH

**Fig 1:**
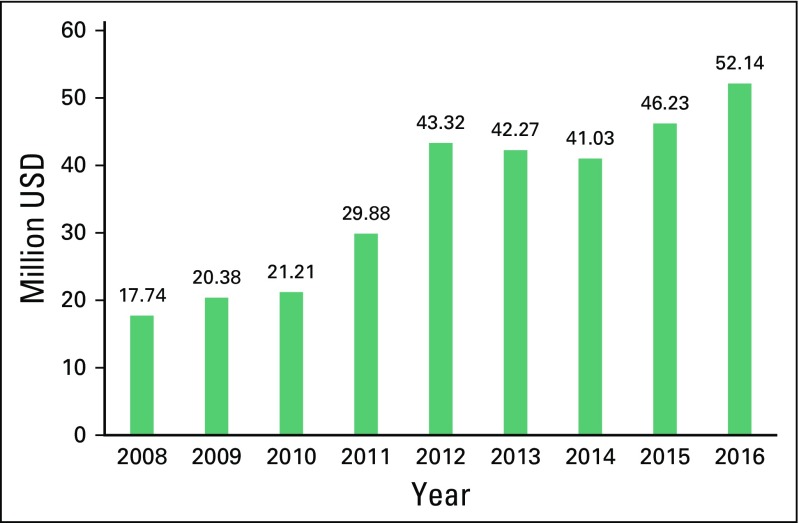
Cost of cancer drugs procured by the Lebanese Ministry of Public Health from 2008 to 2016.

**Fig 2:**
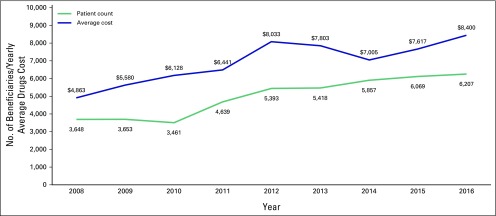
Number of patients and yearly average drugs cost from 2008 to 2016.

Over the 3-year period, a total of approximately $140 million was spent by the MOPH free cancer drug dispensing program. Even though the most expensive cancer drug (trastuzumab, bevacizumab, imatinib, and rituximab) prices were decreased by an average of 35% in 2016 through tough procurement negotiations, the amount spent was slightly over $52 million in 2016 compared with $41 million in 2014. This increase of almost 27% followed the phasing in of immunotherapy drugs. Figure 1 shows trends in drug expenditures using data available for analysis since 2008, with increases linked to the introduction of the newer, more expensive drugs. The number of patients covered by the free cancer drug dispensing program was relatively stable at approximately 6,000 each year, increasing from 5,857 patients in 2014 to 6,207 patients in 2016. Consequently, the average drug cost per patient, measured across all cancer types, increased from $7,000 in 2014 to $8,400 in 2016 (Fig 2).

### Costs by Type of Drug

As in previous years, trastuzumab (Herceptin), which is approved by MOPH for treating human epidermal growth factor receptor 2 (HER2)–positive breast cancer, ranked first in total expenditures for 2014 and 2015. Imatinib (Glivec), which is used for treating chronic myeloid leukemia, was in second place in both 2014 and 2015. New immunotherapy drugs for lung cancer approved in mid-2015 topped the list in 2016: pembrolizumab ranked first and nivolumab ranked third, and $6.5 million was spent on their procurement, which represents almost 64% of the total cost of lung cancer treatment. Table 1 ranks the top 5 most expensive drugs in terms of total yearly spending.

**Table 1:**
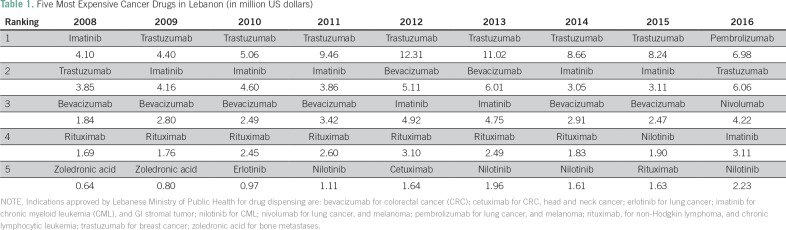
Five Most Expensive Cancer Drugs in Lebanon (in million US dollars)

### Changes in the Cost of Lung Cancer Treatment

According to the latest data from the National Cancer Registry (2015), lung cancer is the second most common cancer type among males (13.1%) and third most common cancer type among females (5.9%).^2^ In 2016, the two immunotherapy drugs (pembrolizumab and nivolumab) accounted for approximately 19% of the total yearly expenditures on cancer drugs. The beneficiaries were 192 patients (24%) of a total of 797 patients with lung cancer. This group of patients represents only 3% of all cancer beneficiaries. The total cost of drugs for treating lung cancer after the introduction of immunotherapy changed, as expected.^6^ It increased from approximately $3 million in 2012 to approximately $10 million in 2016. The average annual cost of drugs per patient with lung cancer increased by 160% between 2012 ($5,000) and 2016 ($13,000).

## DISCUSSION

These new figures document the continuously increasing financial impact of newer cancer drugs on the procurement process in the middle-income country of Lebanon. The increase signals a major financial problem in several developing and developed nations. In the United States, Medicare (the national health insurance program for older adults) spending on Part B drugs (the category dominated by cancer treatment drugs) increased from $3 billion in 1997 to $25.7 billion in 2015 (8.6 times), whereas overall Medicare spending increased from $210 billion to $638 billion (3 times).^7,8^

In Lebanon in particular, the doubling of total costs of oncology drugs over a short 5-year span could not have come at a more difficult time. Since 2011, Lebanon has been facing an influx of refugees from civil wars in Syria and Iraq, which inflated its population by at least 30% for an almost stable population of Lebanese nationals of approximately 4.3 million people.^9^ The tremendous costs of treatment have meant that those refugees previously diagnosed with cancer or diagnosed while in Lebanon could not be easily integrated into the free cancer drug dispensing circuits currently serving only Lebanese patients. The resulting human suffering is putting serious strains on practitioners who find themselves unable to treat those patients adequately.

Although many new cancer therapies have changed the course of the disease, Mailankody and Prasad^10^ reported in a research letter, based on data from the United States, that an independent relationship exists between the price of cancer drugs and their impact on patients’ health; they concluded that the irrational pricing mechanism is driven mainly by market dynamics. Moreover, a recent publication by Kumar et al^11^ showed that only 19% of cancer drugs approved by the US Food and Drug Administration met the ASCO goal of achieving significant clinical outcomes in terms of overall survival, even though the prices are extremely high in many cases. There is an urgent global need for pharmaceutical companies to be held accountable for their pricing practices and to acknowledge their responsibility for human rights.^12^ Disclosure of all costs related to drug discovery, research and development, and marketing would allow transparency and accurate evaluation of the price of a drug in the market compared with the costs needed for its existence. Moreover, international scientific societies are invited to assess the end points of cancer clinical trials to move toward value-based cancer care.^13^

**Fig 3:**
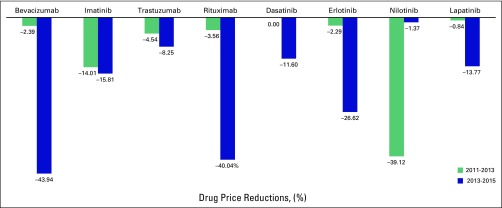
Drug price reductions for selected cancer drugs in Lebanon from 2011 to 2015.

This article is yet another plea for an international approach to limiting the relentlessly increasing costs of cancer drugs that are generating inequity especially in low-income nations where the affordability of both brand-name and generic drugs is lower.^14,15^ The experience in Lebanon shows that measures by one country to decrease the prices of cancer drugs usually have insignificant effects unless they came from the experiences of other countries. Lebanon, a country with a small market, has been able to successfully reduce the prices of several oncology drugs by comparing local prices with those in other countries to generate evidence for negotiating possible discounts. The MOPH established regulations in 2015 requiring that pharmaceutical companies disclose the reduction of any export price in comparable countries within a 3-month interval. The effects of these policies on the prices of selected cancer drugs are shown in Figure 3. The MOPH has recently started to negotiate the price of oncology drugs with drugs companies even before deciding to provide them free of charge to eligible patients. Inspired by the United Kingdom’s National Institute for Health and Care Excellence conducting a cost-effectiveness analysis to include a new drug in its guidelines, and the recent United Kingdom’s National Health Services policy of renegotiating prices to make them more affordable,^16^ the MOPH is reconsidering its policy to ensure the financial sustainability of its free cancer drug dispensing program.

Despite all that, the MOPH is still struggling with high demand for brand-name drugs, even though there are generic competitors or biosimilars. Although these alternatives would improve access to cancer treatment,^17^ decision makers need to understand their importance and not be lenient toward the preferences of providers and manufacturers of original drugs, regardless of their prices. Affordability of cancer treatment should be at the center of decision making for oncology professionals as well. The ASCO position statement on this matter clearly includes drug prices in the core of value-based cancer care and calls upon physicians for appropriate use of oncology drugs, taking into consideration both clinical and financial perspectives.^13^ The experience from Lebanon endorses this suggestion because the common practice in prescribing cancer drugs is somehow driven by financial incentives for health care providers, not only by clinically meaningful outcomes, according to regularly updated national guidelines.^18^

In conclusion, tackling the financial burden of cancer in Lebanon cannot be only based on the cost containment of the procurement of drugs and the subsidization of health services. Initiatives toward primary prevention such as tobacco control, secondary prevention such as screening and early detection, and tertiary prevention by improving the quality of palliative care should be continuously supported. The national tobacco control law, created in 2011, urgently needs to be re-enforced, especially by implementing the portion of that law that bans smoking in enclosed public places. Screening for breast cancer (initiated in 2002) and screening for colorectal cancer (expected in 2018) must be continuously supported by policymakers as being cost-effective. Finally, escalating palliative care initiatives in health facilities in Lebanon would help reduce the use of treatment regimens at the end of life and result in better quality of life for patients with cancer.
